# Probing Membrane Protein Assembly into Nanodiscs by In Situ Dynamic Light Scattering: A_2A_ Receptor as a Case Study

**DOI:** 10.3390/biology9110400

**Published:** 2020-11-13

**Authors:** Rosana I. Reis, Isabel Moraes

**Affiliations:** 1 National Physical Laboratory, Hampton Road, Teddington TW11 0LW, UK; rosana.thevenot@npl.co.uk; 2 Research Complex at Harwell Rutherford, Appleton Laboratory, Harwell, Oxford, Didcot OX11 0FA, UK

**Keywords:** lipids, nanodiscs, adenosine A_2A_ receptor, dynamic light scattering (DLS), size exclusion chromatography (SEC)

## Abstract

**Simple Summary:**

Located within the biological cell membranes, integral membrane proteins are responsible for a large variety of vital cellular processes. In humans, nearly a quarter of the genome codes integral membrane proteins, therefore malfunction of these proteins is associated with a variety of symptoms and diseases such as obesity, cancer and Parkinson’s disease. Clearly, knowledge of membrane proteins behaviour, in both structural and functional terms, is important not only in medicine but also in the design of better drugs with improved pharmaceutical properties. Nevertheless, much still remains unknown about these proteins, mainly because of the technical challenges associated with their production and stability in vitro once removed from their native lipidic environment. Recently, several membrane mimetic systems have been developed including nanodisc lipid particles. Nanodiscs are self-assembled lipidic structures that “trap” membrane proteins into a disc shaped phospholipid bilayer that is stabilised by a belt made of a protein know as membrane scaffold protein (MSP). Membrane proteins assembled into lipidic nanodiscs can maintain their structural and functional integrity and are compatible with most biophysical methods. Here we demonstrate the use of in situ dynamic light scattering as a high-throughput screening tool to assess the best conditions for nanodisc assembly and protein incorporation.

**Abstract:**

Membrane proteins play a crucial role in cell physiology by participating in a variety of essential processes such as transport, signal transduction and cell communication. Hence, understanding their structure–function relationship is vital for the improvement of therapeutic treatments. Over the last decade, based on the development of detergents, amphipoles and styrene maleic-acid lipid particles (SMALPs), remarkable accomplishments have been made in the field of membrane protein structural biology. Nevertheless, there are still many drawbacks associated with protein–detergent complexes, depending on the protein in study or experimental application. Recently, newly developed membrane mimetic systems have become very popular for allowing a structural and functional characterisation of membrane proteins in vitro. The nanodisc technology is one such valuable tool, which provides a more native-like membrane environment than detergent micelles or liposomes. In addition, it is also compatible with many biophysical and biochemical methods. Here we describe the use of in situ dynamic light scattering to accurately and rapidly probe membrane proteins’ reconstitution into nanodiscs. The adenosine type 2A receptor (A_2A_R) was used as a case study.

## 1. Introduction

In the human genome up to 30% of all open reading frames are predicted to encode membrane proteins. These proteins play a crucial role in cell physiology by participating in a variety of fundamental processes such as signal transduction, transport in and out cells, energy conversion and cell communication [1]. Today, more than 50% of the available drugs on the market target membrane proteins highlighting their significance for therapeutic treatments. Therefore, a clear understanding of the structure–function relationships of membrane proteins along with their dynamic mechanisms at cellular and molecular levels is vital to both medicine and early drug discovery [2,3]. Embedded in the cell and organelle membranes and constantly surrounded by different types of lipids in a fluid environment, membrane proteins undergo conformational changes to perform their designated functions. Although lipids are not covalently bound to the proteins, in recent years it has been found that they have a direct impact on the structural folding, assembly and function of membrane proteins. As a result, immense efforts have been made to understand protein–lipid intricate interactions [4,5]. Recent advances in biophysical approaches such as X-ray crystallography, nuclear magnetic resonance (NMR), mass spectrometry and fluorescent methods have provided the first insights into the protein–lipid interactions. Currently, three types of protein–lipid interactions are known: (i) annular shell interaction, where lipid molecules surround the transmembrane domain of the protein surface mediating between the protein and the bilayer membrane; (ii) nonannular interaction, where lipid molecules interact in cavities and clefts present in the protein surface appearing to play key roles in protein multimeric assemblies; (iii) protein–lipid interactions where lipid molecules reside within the membrane protein structure. These are usually found in uncommon positions and are believed to play an important role in membrane protein folding and assembly [6,7].

However, the study of membranes proteins in vitro is far from being trivial. There are still many limitations due to challenges in obtaining high yields of pure and stable protein samples in the presence of native lipids. In general, membrane proteins suffer delipidation as a result of their extraction (or solubilisation) from their native membranes by harsh detergents and purification procedures. Although there is a wide variety of detergents available on the market with different combinations of head and tail groups [8,9], the search for a detergent that can maintain protein structure and function integrity can be a time-consuming and expensive task. Besides, a high concentration of detergent usually results in denaturation of the hydrophobic binding domains and/or occlusion of binding sites in addition to the fact that some binding partners are sensitive to even mild detergent concentration [10,11]. The loss of protein–lipid and lipid–lipid interactions in the micellar environment makes membrane proteins prone to destabilisation. The main reason is because the detergent shell micelle is not able to sustain the structural integrity of the membrane protein to the same extent as its native lipid environment. To circumvent this problem several membrane mimetic systems suitable for investigation of protein structure, dynamics and lipid interplay in a controlled environment have been developed [12,13].

The nanodisc technology is particularly attractive for both structural and functional studies of membrane proteins as it is easily applied to a variety of techniques, such as cryo electron microscopy (cryo-EM), NMR and surface plasmon resonance (SPR) [14,15,16]. Nanodiscs are discoidal structures where the target membrane protein is embedded in a phospholipid bilayer wrapped by two molecules of a protein known as membrane scaffold protein (MSP). MSPs are modified versions of the human apolipoprotein A-1 that are recombinantly expressed in bacteria. Different lengths of MSP constructs allow the production of nanodiscs of different sizes [17,18]. However, the size of the transmembrane portion to be accommodated within the disc and the number of membrane proteins to be incorporated should be considered when determining the ideal size of the disc. The lipid composition surrounding the membrane protein depends on the native cell type, but the most common lipids used in nanodisc technology are zwitterionic (such as 1-palmitoyl-2-oleoyl-sn-glycero-3-phosphocholine (POPC) and 1,2-Dimyristoyl-sn-glycero-3-phosphocholine (DMPC)), negatively charged (such as 1-palmitoyl-2-oleoyl-sn-glycero-3-phospho-L-serine (POPS) and 1-palmitoyl-2-oleoyl-sn-glycero-3-phospho-(1′-rac-glycerol) (POPG)), or a mix of both [19,20,21]. Porcine brain polar lipid extract alone or combined with POPC and POPG have also been used [22,23]. Key factors to be considered when preparing lipidic nanodiscs include: (i) purity/stability of the target membrane protein, (ii) MSP:target protein ratio and (iii) MSP:lipid ratio. Unfortunately, the ratio between lipids mixtures, MSPs and lipids and MSP and target protein needs to be determined empirically. An incorrect ratio leads to the irreversible aggregation of either target protein or MSP or formation of bare discs [17]. Finally, the ratio combination needs to be adjusted for each new protein target in study.

In this study, we show the practicality of in situ Dynamic Light Scattering (DLS) compared with conventional DLS methods to probe successful incorporation of integral membrane proteins into lipidic nanodisc particles. In contrast to conventional DLS systems, the in situ DLS approach allows serial measurements in parallel by using multi-well crystallisation batch plates (standard SBS plates or Terasaki microbatch plates), requiring only very small sample volumes (0.5 to 2 μL) at very low protein concentration (from 0.3 mg/mL). The system, that also has a built-in microscope, allows sample drops to be visualised (e.g., to check air bubbles) prior to measurements. Samples are also kept at a constant temperature by the instrument’s temperature control unit. This noninvasive high-throughput approach proves to be a fast and reliable tool to screen large number of MSP:lipid and protein:MSP:lipid ratio conditions when looking for the best mixture for the protein-nanodisc assembly. The system contains several analytical tools (displayed on a user-friendly interface) such as size distribution plots in the form of signal heat map, graphs of the autocorrelation function and radial distribution plots that help fast visualisation and analysis of the measurement results. Finally, the in situ DLS approach also comes with the advantage of being able to perform time-resolved measurements, if required [24,25].

The protein used in this study, the Adenosine type 2A receptor (A_2A_R), belongs to the largest superfamily of integral membrane proteins in the human genome known as G protein-coupled receptors (GPCRs). These receptors all share the same structural topology of seven transmembrane α-helices comprising an extracellular N-terminus and an intracellular C-terminus. The G protein-coupled adenosine receptor family belongs to the GPCR class A (rhodopsin-like receptors) and it is divided in four subtypes—A1, A_2A_, A2B and A3 [26]. Adenosine receptors are largely expressed in the central nervous system as well as in cardiovascular, respiratory and renal tissues including the immune system. As a result, they are implicated in a wide range of pathophysiological conditions such as Parkinson’s disease, dementia, arrhythmia, asthma, type 2 diabetes, glaucoma, inflammation and cancer [27,28,29,30]. The A_2A_R in particular is involved in the activation of the G_αs_ family member and is thus implicated in sleep, angiogenesis and immunosuppression regulation [26]. Despite numerous crystal structures of the A_2A_R having been solved in a complex with antagonists, agonists and G_s_ protein, much is still unknown regarding its mechanism of action. Recently, molecular dynamics simulations studies have suggested that different membrane phospholipid types could lead to different A_2A_R conformational states that in turn result in different functional responses [31]. Therefore, based on the importance of the A_2A_R as a drug target, the influence that different endogenous lipids might have in A_2A_R responses and its structural features as an integral membrane protein, we found the A_2A_R to be a good candidate for our case study.

## 2. Materials and Methods

*N*-Decyl-β-d-maltopyranoside (DM) was purchased from Anatrace (Maumee, OH, USA) sodium cholate and BioBeads were purchased from Merck (Dorset, UK). 1-Palmitoyl-2-oleoyl-sn-glycero-3-phosphocholine (POPC), 1-palmitoyl-2-oleoyl-sn-glycero-3-phospho-(1′-rac-glycerol) (POPG) and porcine brain polar lipids (BPLs) were from Avanti Polar Lipids (Alabaster, AL, USA). All other reagents were analytical grade.

### 2.1. Protein Expression and Purification

#### 2.1.1. A_2A_R Purification

**Figure 1:**
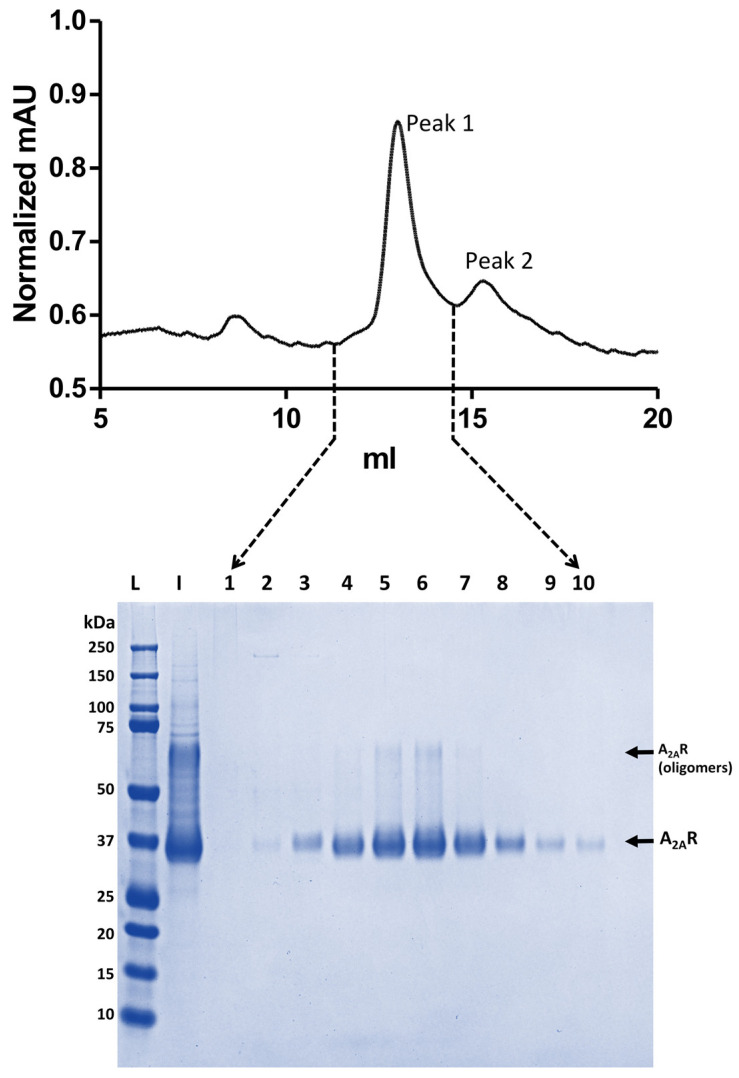
Size exclusion chromatography profile of the detergent solubilised adenosine type 2A receptor (A_2A_R) in 0.15% *N*-Decyl-β-d-maltopyranoside (DM) and sodium dodecyl sulphate-polyacrylamide gel electrophoresis (SDS PAGE) analysis of the eluted protein fractions. Chromatogram peak 1 represents the purified protein and peak 2 the UV absorbance of the free A_2A_R antagonist ligand (theophylline). The higher molecular weight (~75 kDa) band in SDS PAGE gel corresponds most probably to SDS-resistant A_2A_R dimers. Abbreviations used: L, ladder; I, sample loaded into the column; 1 to 10, eluted fractions and kDa, kilodalton.

The A_2A_R construct used has been described previously [32] and it was modified to contain a FLAG tag. A_2A_R was expressed and purified as described in [32] with modifications. Briefly, the receptor was expressed in Sf9 cells using the Bac to Bac Expression System (Invitrogen). Cells were infected at a density of 2 × 10^6^ cells/mL with the virus at an approximate multiplicity of infection of 1. Cultures were grown at 27 °C with constant shaking and harvested 48 h postinfection. Cells pellets were resuspended in buffer consisting of 40 mM Tris buffer pH 7.4 supplemented by 1 mM EDTA and protease inhibitors (Merck, Dorset, UK; Cat. No. 11873580001). After cell disruption by Dounce homogeniser, membranes were pelleted by ultracentrifugation at 100,000× *g* for 40 min. Following this, membranes were subjected to a high salt wash in a buffer containing 40 mM Tris pH 7.4, 1 M NaCl and protease inhibitors and centrifuged at 100,000× *g* for 40 min. Washed membranes were resuspended in 40 mM Tris pH 7.4, protease inhibitors, 3 mM theophylline (Merck, Dorset, UK; Cat. No. T1633-100G) and incubated for 1 h at room temperature with constant mixing. Membranes were then solubilised by the addition of 1.5% n-Decyl-β-d-maltopyranoside (DM, Anatrace), and incubation for 2 h at 4 °C, followed by centrifugation at 100,000× *g* for 1 h. The solubilised material was filtered and incubated with Ni-NTA resin (Thermo Fisher, Waltham, MA, USA) pre-equilibrated in 40 mM Tris pH 7.4, 200 mM NaCl, 0.15% DM and 1 mM theophylline. The resin was washed with 40 column volumes of 40 mM Tris pH 7.4, 200 mM NaCl, 0.15% DM, 70 mM imidazole and 1 mM theophylline and then the protein was eluted with 40 mM Tris pH 7.4, 200 mM NaCl, 0.15% DM, 280 mM imidazole and 1 mM theophylline. Collected fractions were analysed by sodium dodecyl sulphate-polyacrylamide gel electrophoresis (SDS PAGE) and fractions containing A_2A_R were pooled and applied to a Superdex 200 Increase 10/300GL size exclusion column (GE Healthcare) pre-equilibrated with 40 mM Tris pH 7.4, 200 mM NaCl, 0.15% DM and 1 mM theophylline. Eluted fractions containing the A_2A_ protein were analysed by SDS PAGE (Figure 1), pooled and concentrated to ~20 mg/mL.

#### 2.1.2. MSP1D1 Purification

The Membrane Scaffold Protein 1D1 (MSP1D1) construct was obtained from Addgene (Addgene plasmid #20061) [33]. The protein was expressed and purified according to [34] with modifications. Briefly, the protein was expressed at 37 °C using BL21(DE3) *E. coli* cells (Calbiochem) in Terrific Broth (TB) medium until the OD600 reached 1.6. Expression was induced with 1 mM IPTG and the cells were harvested by centrifugation (8000× *g*, 4 °C, 15 min). Cells were disrupted by sonication in 50 mM Tris pH 7.5, 300 mM NaCl and protease inhibitors (Roche). The supernatant was cleared by centrifugation at 30,000× *g* at 4 °C for 1 h. The latter was purified using a His-Trap column at 4 °C. The column was washed with 20 column volumes of 50 mM Tris pH 7.5, 300 mM NaCl and 10 mM imidazole, and the protein was eluted in 50 mM Tris pH 7.5, 300 mM NaCl and 280 mM imidazole. Imidazole was removed by dialysis, and protein was concentrated using a 10 KDa MW cut off concentrator to 10–15 mg/mL.

### 2.2. A_2A_R Reconstitution into Nanodiscs

**Figure 2:**
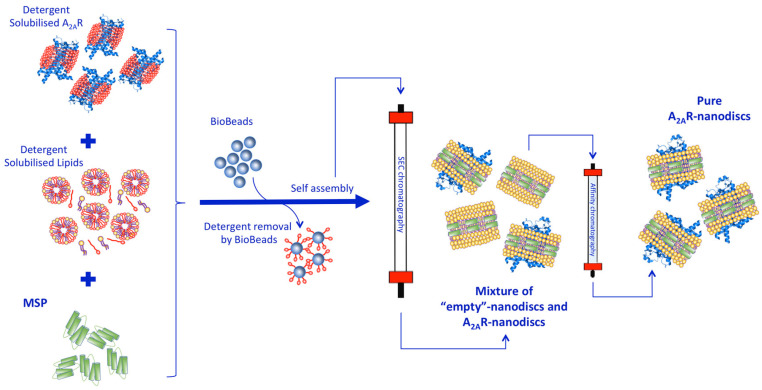
Cartoon representation of a standard nanodisc assembly protocol. Detergent-solubilised membrane protein (A_2A_R) is incubated with the membrane scaffold protein (MSP) and the lipid–detergent mixture at the target concentration ratio. Following, Biobeads are added to the mixture to remove detergent and initiate nanodiscs’ assembly. Traditionally, size exclusion chromatography (SEC) is performed to assess the disc formation and if successful, affinity chromatography is executed to remove the nanodisc particles that are bare. Detergent molecules are represented in red, lipids in yellow, protein receptor in blue and membrane scaffold protein in green.

Protocols for reconstitution of membrane proteins into nanodiscs were followed as reported in [34,35,36,37,38] (Figure 2). However, optimal ratios of MSP:A_2A_R and MSP:lipids were empirically determined. Two different types of lipid composition, porcine brain polar lipid (BPL) and a 1:1 molar ratio of POPC:POPG lipid mixture were used for the reconstitution of A_2A_R into nanodiscs. While purified MSP and A_2A_R were mixed at an 80:1 molar ratio, respectively, in 40 mM Tris pH 7.4 and 200 mM NaCl, the MSP:lipid mixture ratio was 1 to 50, 60 and 80 depending on the lipid used. The MSP:A_2A_R:lipid samples were incubated for 1 h on a rotating wheel at 4 °C. Subsequently, BioBeads were added to the samples (for detergent removal) and left overnight again on a rotating wheel at 4 °C. The BioBeads were separated from the assembly using centrifugation at 3000× *g* and any additional precipitate in the solution was removed by further centrifugation at 14,000× *g* for 15 min. Samples were then subjected to in situ DLS measurements and size exclusion chromatography (SEC).

### 2.3. In Situ DLS Measurements

The size distribution of the nanodisc samples was analysed using the recently developed in situ DLS system SpectroLight 610 from XtalConcepts GmbH (Hamburg, Germany). The SpectroLight 610 DLS system is equipped with a 100-mW laser diode (λ = 660 nm, red), a sensitive detector positioned at a scattering angle of 142° and a multi-tau architecture correlator. The DLS SpectroLight 610 system software includes a Laboratory Information Management System (LIMS) that stores the acquired data in an SQL database. It also contains several analytical tools that are accessible and displayed on the user-friendly interface. The samples were spun in a top bench centrifuge at 14,000× *g* for 10 min prior to measurements. A volume of 2 µL of each sample was loaded onto 72-well Terasaki microbatch plates (Molecular Dimensions) covered with paraffin oil (Fisher). Prior to measurements, sample drops were visualised by the SpectroLight 610 system’s built-in microscope for checking air bubbles and verification of the drop centring. Measurements were kept at a constant temperature of 20 °C by the instrument’s temperature control unit. The scattered light photon signals were processed using the in situ DLS system software and converted into various types of plot that include the autocorrelation function, radius distribution and radial distribution for analysis.

### 2.4. Size Exclusion Chromatography

SEC of the assembled nanodiscs was carried out using a mobile phase consisting of 40 mM Tris pH 7.4, 100 mM NaCl, 5 mM MgCl_2_ and 1 mM theophylline on a Äkta Purifier FPLC system (GE Healthcare, Chicago, IL, USA) using coupled to a Superdex 200 Increase 10/300 GL column (GE Healthcare, Chicago, IL, USA) at 1.8 and 1.9 MPa and flow rate of 0.5 mL/min. The absorbance of the eluate was measured at a wavelength of 280 nm.

## 3. Results

### 3.1. Assessing A_2A_R-BPL Nanodisc Formation

**Figure 3:**
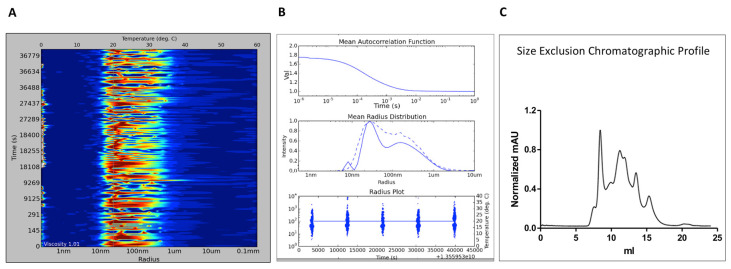
In situ Dynamic Light Scattering (DLS) analysis of A_2A_R-BPL nanodiscs. The A_2A_R:MSP ratio was 1:80 and the MSP:lipid ratio 1:80. The panel (**A**) shows the size distribution plot in the form of signal heat map while (**B**) shows the DLS in situ analysis through the graphs of the autocorrelation function, radius distribution and radial distribution plot (the blue spot diameter represents the relative scattered light intensity of the detected particles in arbitrary units). Normalised SEC chromatogram is shown in (**C**).

As A_2A_R is widely expressed in the central nervous system, we thought that BPL would be a good candidate for probing nanodisc formation. BPL is derived from total lipid extract and its major lipid component is phosphatidylethanolamine (PE). PE lipids have been shown to be important for the activation of rhodopsin, NTS1 and μ-Opioid receptors [23,39]. BPL also contains phosphatidic acid and phosphatidylinositol. From our experience, nanodisc formation using this type of lipid is particularly challenging with a significant amount of the protein being localised in the aggregate during the conventional SEC analysis. Therefore, we used in situ DLS to rapidly assess A_2A_R-BPL nanodisc assembly and compare it with its SEC profile (Figure 3). While the A_2A_R:MSP ratio used was 1:80, the MSP:lipid ratio was 1:80. In situ DLS results revealed the presence of many particles of different sizes (Figure 3A,B) that were also confirmed by SEC analysis of the sample (Figure 3C). This result clearly indicates that the MSP:BPL ratio of 1:80, although a good ratio for other GPCRs [22], is unsuitable for the A_2A_R reconstitution.

### 3.2. Assessing A2AR-POPC:POPG Nanodisc Formation

**Figure 4:**
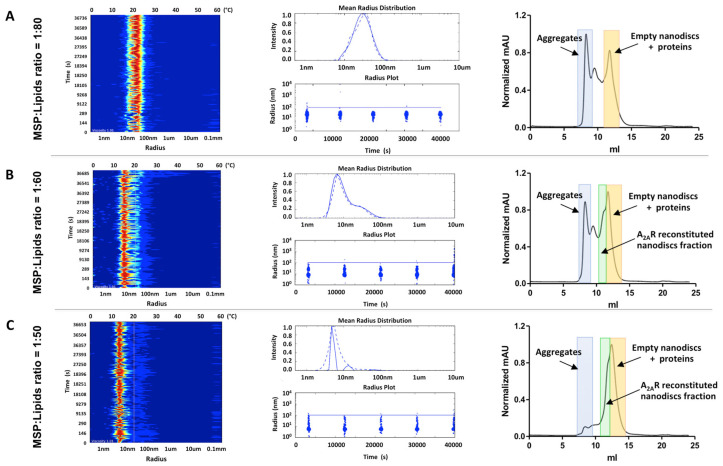
In situ DLS analysis of three A_2A_R-POCP: POPG nanodisc samples produced with different MSP:lipid ratios. The MSP:lipid ratios were 1:80 (**A**), 1:60 (**B**) and 1:50 (**C**). The left panels show the size distribution plot in the form of a signal heat map followed by the in situ analysis panel showing the graphs of the radius distribution and radial distribution plot (the blue spot diameter represents the relative scattered light intensity of the detected particles in arbitrary units) for each of the ratios. The corresponding SEC chromatograms for each MSP:lipid nanodisc samples are shown on the right hand side.

The reconstitution of A_2A_R into POPC: POPG nanodiscs was performed with an 80-fold molar excess of MSP to A_2A_R to favour reconstitution of a single A_2A_R molecule per disc. As the ratio between MSP and lipids is critical for successful assembly of nanodiscs, here we used three different MSP:lipid ratios 1:80, 1:60 and 1:50 to probe the best reconstitution condition. From the in situ DLS measurements, improvements in nanodisc assembly were immediately observed as the MSP:lipid ratio decreased (Figure 4). At MSP:lipid 1:80 molar ratio, although nanodiscs were formed, large amounts of aggregates were also present as indicated by the in situ DLS radius distribution signatures (Figure 4A) and confirmed by the SEC profile. At lower lipid concentrations, as in the case of MSP:lipid at a 1:60 molar ratio, less aggregation was observed; however, the sample could not yet be considered monodispersed (Figure 4C). Finally, for a molar ratio of MSP:lipid at 1:50, a monodisperse profile was observed by in situ DLS measurements and SEC profile (Figure 4C) indicating that this is the best of the conditions tested for A_2A_R-POPC:POPG nanodisc formation.

## 4. Discussion

Despite the popularity of nanodisc technology among the membrane protein research community, incorporation of membrane proteins into lipidic nanodiscs is still hindered by the large number of reconstitution conditions that need to be tested. In general, the starting point is the information available in the literature (as we did in this study), but ultimately a variety of phospholipids’ compositions and different molar ratios between the membrane protein in study, MSPs and lipids always need to be experimentally tested until the ideal reconstitution condition is found. The traditional approach is to use SEC to assess protein–nanodisc assembly for each of the MSP:lipid and MSP:membrane protein ratio conditions (Figure 2). This is laborious and requires large amounts of buffers, protein and lipids, depending on many ratio conditions one is screening.

**Figure 5:**
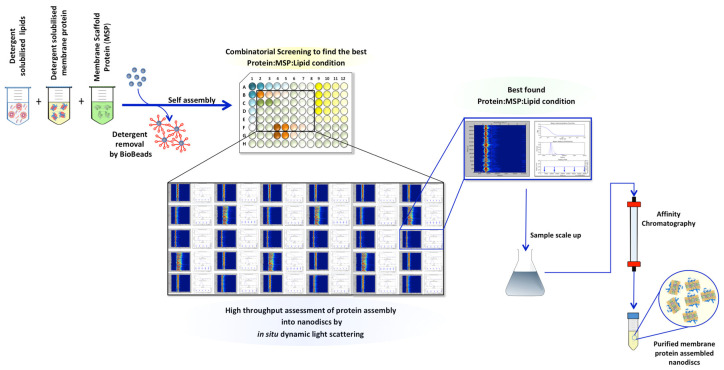
Schematic representation of the membrane protein assembly into nanodiscs process using in situ DLS as a high-throughput analytical tool to probe best protein:MSP:lipid condition.

Although conventional DLS measurements have previously been used with success [18,40,41], here we introduce the recent developed in situ DLS system as a high-throughput analytical tool to rapidly probe the best reconstitution conditions for membrane protein assembly into nanodiscs (Figure 5). While conventional DLS systems usually require large amounts of samples, extensive cuvette cleaning and only one sample per measurement, in situ DLS approach which is fully automated, is able to measure up to 72 samples in parallel using only microliter volumes of sample per measurement. As proof of concept, in this study we used a thermostabilised human A_2A_ receptor as a membrane protein test case. For nanodisc assembly, BPL as well a ratio mixture of POPC:POPG lipids in the presence of the same MSP construct (MSP1D) were used. Four different conditions (around known conditions from the literature) were assessed in parallel by in situ DLS measurements and cross-validated by SEC. Although the results obtained from the in situ DLS correlated well with the SEC profiles, there was an obvious difference between the two methods. While only minutes were needed to assess the best MSP:A_2A_R:lipid nanodisc formation profile, it took days to collect the SEC results. Moreover, the amount of A_2A_R, MSP and lipids used were much higher during the SEC experiments.

Automation, miniaturisation, and even integration have played a critical role in research. Reducing the number of repetitive manual tasks, in our case here, several SEC runs or individual DLS measurements, considerably decreases potential for error. In addition, it would also significantly increase savings in terms of time and costs. This allows researchers to concentrate on research rather than repetition. In our study, we only tested four different conditions regarding to lipid composition and MSP:lipid ratio; however, with the use of in situ DLS as a high-throughput analytical tool, many other parameters could be tested such as stability of the protein in study, different construct lengths of the MSP to probe different nanodisc sizes and stability of the newly assembled nanodiscs over time.

In summary, our study and results not only stress the importance of high-throughput approaches in modern lipidic nanodisc technology, but also the importance of developing and adopting new strategies that fast find the best nanodisc assembly protocol. Moreover, high-quality protein–nanodisc particles will significantly improve the data collection quality on the downstream biophysical applications such as cryo-EM, NMR and SPR.

## 5. Conclusions

In recent years, the use of lipidic nanodisc technology for membrane protein studies became extraordinarily popular as it provides a more native-like membrane environment than detergent micelles or liposomes [17,42]. In addition, it is compatible with a variety of biochemical and biophysical approaches [14,15,16,43,44]. Advantages in the use of nanodisc systems include better control of membrane protein solubility, oligomerisation state and improved stability and functionality. However, when preparing nanodiscs for membrane protein studies, lipid composition and stoichiometry between MSP, protein target and lipids is crucial for successful nanodisc formation and membrane protein assembly [18,45]. However, these need to be empirically determined and changed for every different protein target in study.

Our results show that in situ DLS is a valuable tool when screening and optimizing conditions for membrane protein nanodisc assembly. In situ DLS allows the use of multi-well plates to screen different ratio conditions in parallel within minutes. The low protein concentration and small volume required, when using the in situ DLS approach, allow researchers to test a variety of different conditions until the best one is found. The radius distribution signatures and “heat maps” from the in situ DLS analytical tools allow direct and fast monitoring of the protein-nanodisc assembly without the need of performing size exclusion chromatography for each of the different MSP:lipid ratio conditions. Thereby, the approach reported here provides a cost-effective platform for fast results and productivity regarding to membrane protein studies in native-like environments such as lipidic nanodiscs.
